# Redesigning Prison Healthcare: Operational Insights and Leadership Lessons from a Correctional Medical Center

**DOI:** 10.3390/healthcare14213800

**Published:** 2026-11-06

**Authors:** Giulio Di Mizio, Francesca Bruno, Valentina Pugliese, Laura Iozzo, Giuseppe Cundò, Raffaele Gaetano, Vito Barbieri, Raffaele Maio, Luca Gallelli

**Affiliations:** 1 Prison Medical Center, Intensive Assistance Service (SAI), Catanzaro Health Authority “U. Caridi” Correctional Center (Italian Ministry of Justice), 88100 Catanzaro, Italy; 2 Forensic Medicine and Criminology, Law Department, University Campus “S. Venuta”, 88100 Catanzaro, Italy; 3 Oncology Unit, Oncology Department, “Azienda Ospedaliero Universitaria (AOU) Dulbecco” Hospital, 88100 Catanzaro, Italy; 4 Geriatrics Unit, P.O. Germaneto, A.O.U. “R. Dulbecco”, 88100 Catanzaro, Italy; 5 Department of Health Sciences, Magna Graecia University of Catanzaro, 88100 Catanzaro, Italy; gallelli@unicz.it; 6 Operative Unit of Clinical Pharmacology and Pharmacovigilance, “R. Dulbecco” University Hospital, Viale Europa, 88100 Catanzaro, Italy

**Keywords:** penitentiary medicine, clinical governance, risk management, informed consent, therapeutic alliance, healthcare quality, prisoner health

## Abstract

Correctional healthcare is delivered at the intersection of two institutional mandates: protection of health and maintenance of custody and security. This commentary develops an experience-based operational re-engineering model for prison healthcare, building on the Italian transition of penitentiary medicine to the National Health Service (NHS) and on direct executive management within a Prison Medical Center. Rather than proposing a new regulatory architecture, the model translates existing legal, ethical, and clinical-governance requirements into operational processes. Particular attention is given to the practical implementation of Article 11 of the Italian Penitentiary Law, equivalence of care and Essential Levels of Care, informed consent and treatment refusal, clinical risk management, medication governance, electronic traceability, dedicated clinical pathways, telemedicine, gender-responsive care, and continuity at transfer or release. The central organizational premise is that the healthcare-custody interface should be governed through shared procedures, explicit responsibilities, and auditable clinical documentation, while preserving clinical independence and patient autonomy. The proposed framework is intended as an adaptable, experience-informed operational model that may support improvements in safety, appropriateness, continuity, accountability, and equity in correctional healthcare, including in other jurisdictions.

## 1. Introduction

### 1.1. Background: Conceptual Link to the 2021 Discussion

In 2021, the question “Health in Prison: Does Penitentiary Medicine in Italy Still Exist?” was used to examine the fragmented historical and regulatory evolution of Italian prison healthcare [1]. The subsequent policy trajectory confirms that the central problem is no longer the formal recognition of the right to healthcare but its consistent operational implementation. The legal basis of Italian prison healthcare is now well established: equivalence of care was introduced in 1999, prison healthcare was transferred to the NHS in 2008, and the relationship between prison health services, regional networks, and external services was defined in 2015 [2,3,4,5,6], in line with European standards emphasizing integration with national health systems, equivalence, continuity, and non-discrimination [7,8,9]. Nevertheless, formal integration does not automatically eliminate the operational friction generated by the coexistence of healthcare and custodial systems. The clinically oriented logic of the NHS and the security-oriented logic of the Penitentiary Administration remain structurally interdependent. For this reason, the next step for penitentiary medicine is not merely further doctrinal description but the systematic redesign of interfaces, responsibilities, documentation, and clinical pathways.

### 1.2. Perspective from the Direction of a Prison Medical Center

The Diagnostic and Therapeutic Penitentiary Center (formerly SAI) represents a high-intensity node within the correctional healthcare network. It receives people in custody with acute or subacute conditions, advanced chronic diseases, infectious diseases, psychiatric or substance-use comorbidity, disability, or other clinical needs that cannot be adequately managed in ordinary detention sections but do not necessarily require admission to an external hospital. Leadership of such a center provides a distinct organizational perspective because clinical decisions are continuously conditioned by transfers, security procedures, judicial interfaces, staffing, treatment adherence, and continuity of care. In this setting, the director must preserve the independence of clinical judgment while simultaneously coordinating the interfaces that determine whether a clinically appropriate decision can be implemented in practice [6,7,8,9].

- Institutional Integration Hub: The Prison Medical Center functions as an operational interface between the NHS and the Penitentiary Administration. Its role is not to merge healthcare and security responsibilities but to translate clinical priorities into workflows that are feasible within the custodial environment, through agreed procedures, escalation pathways, and clearly assigned responsibilities.
- Medico-Legal and Judicial Coordination Interface: Following the transfer of prison healthcare to the NHS, clinical decision-making remains professionally autonomous; however, some healthcare pathways require interaction with prison management or judicial authorities because movement, external transfer, detention status, and specific procedural safeguards may affect implementation. Accurate documentation and clear distinction between clinical and custodial responsibilities are therefore essential.
- Clinical Governance and Risk Management Setting: Correctional healthcare combines clinical complexity with structural constraints that are uncommon in ordinary outpatient settings. Delayed access, restricted movement, medication diversion, self-harm risk, infectious disease, high mental-health burden, and fragmented transitions all require risk-management systems adapted to the custodial environment rather than simply imported from hospital practice [7,8].

The center in which the model was developed operates 24 h a day, with physicians, nurses, and healthcare assistants always on site and a medical supervisor available by telephone at night, who intervenes in person when required (e.g., riots, intoxications, multiple simultaneous critical events, or severe agitation) and maintains contact with the reference hospital for all hospitalized detainees. The center includes inpatient beds within the prison (Intensive Assistance Service, SAI), two equipped emergency rooms with a continuously available and supervised blood gas analyzer, and a radiology section with digital radiography, orthopantomography, and two ultrasound systems. Specialists in cardiology, infectious diseases, urology, pulmonology, physical medicine and rehabilitation, diabetology, and orthopedics, as well as a nutritionist, are stably present, and the mental health team includes three full-time psychiatrists and eight psychologist-psychotherapists. Blood samples are processed with priority by the nearby university hospital, which can be reached within minutes; this allows, for example, the on-site observation of patients with chest pain when safety criteria are met. Measures already implemented in the center include directly observed therapy, electronic clinical records with electronic prescribing and administration, electrocardiography, bedside ultrasound, multidisciplinary triage, dedicated pathways for hepatitis C and mental health, and regular clinical governance and risk-management audits, including joint audits with physicians of the reference hospital.

### 1.3. Objectives and Scope

This commentary proposes an experience-informed operational re-engineering strategy for correctional healthcare; it is not a systematic review, and it does not claim that a single organizational model is universally applicable. The framework was developed through three interacting sources. First, recurring operational problems were identified through the direct management of the Prison Medical Center, including clinical governance and risk-management audits, interface failures between healthcare and custody, and difficulties in transfers and continuity. Second, each problem was mapped against the obligations established by the Italian regulatory framework, in order to distinguish implementation gaps from gaps in the law itself. Third, the proposed responses were compared with selected international standards and evidence, which were used to test their consistency with equivalence of care and to support their adaptation beyond the local setting. Throughout the text, measures already in place at the authors’ center, problems observed in practice, and recommendations for future development are explicitly distinguished. The model is organized around four objectives:

1. Ensuring Essential Levels of Care (LEAs) and equivalence of care without discrimination related to legal status. Legislative Decree 230/1999, the 2008 transfer to the NHS, the 2015 Unified Conference Agreement, and European standards all support the principle that people in custody should have access to clinically appropriate prevention, diagnosis, and treatment comparable to those available in the community, while recognizing the logistical constraints of detention [2,3,6,7,8,9].
2. Rebuilding the therapeutic alliance between clinicians and patients in custody by maintaining clinical independence, confidentiality, informed consent, and transparent communication. In a coercive environment, distrust may arise when patients perceive clinicians as part of the custodial apparatus or when professionals interpret symptoms primarily through a security lens. Operational separation of healthcare and custodial functions is therefore a prerequisite for credible patient-centered care [8,9,10,11].
3. Reducing avoidable clinical and organizational risk through standardized triage, medication governance, traceable documentation, shared emergency procedures, appropriate use of internal diagnostics, telemedicine, and rational criteria for external transfer.
4. Strengthening continuity of care across internal transfers, external hospital episodes, and release to the community. Continuity should be treated as a core quality indicator because interruptions in treatment disproportionately affect people with mental illness, substance-use disorders, chronic disease, and infectious disease [7,8,12,13].

## 2. Evolution of the Regulatory and Organizational Framework

### 2.1. Institutional Dualism: Healthcare (NHS) vs. Custody (Penitentiary Administration)

Healthcare delivery in Italian correctional institutions is characterized by institutional dualism. The NHS, through regional and local health authorities, is responsible for clinical care according to the principles of universality, equity, appropriateness, and patient safety. The Penitentiary Administration is responsible for custody, security, order, and the broader rehabilitative framework. These mandates are distinct but operationally interdependent. Healthcare professionals generally do not control movement between sections, escort availability, security screening, or transport outside the prison, yet these variables may determine whether a diagnostic or therapeutic plan is delivered on time. The 2015 Unified Conference Agreement explicitly addresses the need to coordinate prison healthcare networks and external services, while European standards require prison health services to remain integrated with national health policy [6,9]. Re-engineering should therefore treat the NHS–custody relationship as a managed interface: clinical decisions remain healthcare decisions, whereas the operational conditions necessary to implement them are addressed through shared Standard Operating Procedures (SOPs), predefined escalation mechanisms, and documented responsibilities.

In daily practice, conflicts between security schedules and clinical timing—for example, when a specialist visit, a diagnostic test, or an urgent transfer coincides with counts, movement restrictions, or a shortage of escort staff—should be managed through a predefined escalation pathway rather than case-by-case negotiation. A pragmatic sequence is as follows: (i) the physician on duty documents the clinical priority and the maximum clinically acceptable delay and communicates them to the custodial officer in charge; (ii) if the conflict persists, the medical supervisor and the prison director, or their delegates, agree on a solution; and (iii) in time-critical emergencies, the emergency response is activated on clinical grounds and custodial arrangements are organized in parallel, not as a precondition. In cases of extreme urgency, the physician on duty certifies the need for immediate external transfer, which does not require prior judicial authorization (see the note to Table 1). Each conflict and its resolution should be recorded and reviewed in governance audits, so that recurring problems lead to changes in shared procedures. In the authors’ center, continuous on-site coverage and an on-call medical supervisor in contact with the reference hospital support this process outside office hours.

### 2.2. Article 11 of the Italian Prison Law: Operational Application

Article 11 of Law 354/1975, as amended by Legislative Decree 123/2018, provides the principal statutory framework for healthcare within Italian prisons [4,5] (Table 1). Together with D.P.R. 230/2000 and the post-2008 NHS framework, it establishes the obligation to provide healthcare within correctional institutions and to activate external pathways when necessary diagnostic or therapeutic interventions cannot be adequately provided internally [3,6,14]. From an operational perspective, the most relevant domains are as follows:

- Medical Screening on Admission and Documentation: Intake assessment should identify acute disease, chronic conditions, infectious risks, mental-health and suicide risk, substance use, current therapy, disability, and evidence of violence or ill treatment. Documentation must be clinically complete, timely, and capable of supporting continuity across subsequent transfers. International standards similarly consider early medical assessment a core component of prison healthcare [8,9].
- Transfers for External Medical Care: When necessary diagnostic or therapeutic interventions cannot be safely provided within the institution, transfer to an external healthcare facility is required. Under Article 11(4) of Law 354/1975, the competent judicial authority varies according to procedural status; however, the clinical indication for transfer remains a healthcare decision and should be documented independently of custodial or procedural authorization [4,5].
- Private Medical Consultations: People in custody may request private medical consultations at their own expense where permitted by the applicable legal framework. Such requests should be managed transparently, with respect for patient autonomy and institutional rules, without creating an additional clinical-necessity threshold beyond that required by law. Healthcare professionals should document the request, clinical information exchanged, and any implications for ongoing treatment.

### 2.3. Essential Levels of Care (LEAs) Behind Bars

The D.P.C.M. of 1 April 2008 and the subsequent Unified Conference Agreement of 22 January 2015 place prison healthcare within the NHS and reinforce access to healthcare pathways consistent with the Essential Levels of Care (LEAs) [3,6]. The principle is also consistent with WHO and Council of Europe standards on equivalence and integration with community healthcare [7,8,9]. The practical challenge is therefore not whether people in custody are entitled to care but how to ensure that equivalent clinical standards remain achievable despite high turnover, transfers, language barriers, security procedures, and variable availability of specialist services.

- Primary and Secondary Prevention: Screening and prevention programmes for infectious diseases, cancer, cardiovascular risk, vaccination, and other conditions should follow national or regional standards while being adapted to the high turnover of the prison population. WHO data indicate that infectious diseases, mental-health conditions, and continuity at transition remain priority domains in European prison health systems [8].
- Specialist and Laboratory Access: Internal specialist capacity varies between institutions and regions. A network-based model should combine scheduled on-site clinics, point-of-care diagnostics where appropriate, teleconsultation, and clearly defined criteria for external referral. The aim is not to minimize external access as an end in itself but to provide the right level of care in the safest and most proportionate setting.
- Prison Health Services Charter: Each institution should provide accessible information on available healthcare services, routes for requesting care, waiting and referral procedures, confidentiality, informed consent, complaints, and continuity arrangements. A transparent service charter supports both health literacy and accountability and should be available in formats suitable for people with language, literacy, or disability-related barriers.

## 3. Re-Engineering the Therapeutic Alliance and Informed Consent

### 3.1. Rebuilding Trust in a Coercive Environment

The clinician–patient relationship in prison develops within a coercive environment but must remain a healthcare relationship. Unlike community care, patients cannot freely choose the setting and may have limited choice of healthcare professionals. At the same time, clinicians may work in close proximity to custodial staff and may be asked for opinions with administrative or judicial consequences. These circumstances create a recognized risk of dual-loyalty tension and may weaken trust if healthcare decisions are perceived as extensions of punishment or security [9,11]. Two symmetrical distortions should be avoided:

- Defensive Clinical Skepticism: Clinicians may become overly suspicious of reported symptoms because requests for care occur in a setting in which movement, work, placement, or external transfer can have non-clinical consequences. Such contextual awareness is legitimate, but it should not replace objective clinical assessment or evidence-based decision-making.
- Instrumental Use of Healthcare: In some cases, healthcare requests, medication, self-injury, or treatment refusal may be influenced by motives that extend beyond disease management. These possibilities should be assessed case by case rather than generalized to the prison population. The appropriate response is structured clinical assessment, risk stratification, multidisciplinary review when indicated, and consistent documentation.

Operational re-engineering of the therapeutic alliance therefore requires clinicians to avoid both therapeutic naivety and automatic suspicion. Behaviors such as symptom amplification, foreign-body ingestion, medication diversion, or self-harm can occur in custodial settings, but they may reflect heterogeneous drivers including psychiatric illness, distress, substance dependence, coercion, interpersonal conflict, or attempts to alter custodial circumstances. Risk management should focus on the individual clinical mechanism and not on presumed intent alone. Neutrality is strengthened through the following:

- Clinical Standard Invariance: Diagnostic and therapeutic decisions should follow the same evidence-based standards, professional duties, and patient-safety principles that apply elsewhere in the NHS, with contextual adaptation only where the custodial environment materially affects implementation [8,9,15].
- Communicative Transparency: Patients should receive clear information on what is clinically indicated, what can be provided internally, when external care is required, what delays are clinically acceptable, and which aspects of the process are controlled by healthcare versus custodial authorities. This distinction helps preserve trust and reduces the risk that healthcare decisions are interpreted as punitive or negotiable.

### 3.2. Implementing Law 219/2017: Patient Autonomy in Custody

Law 219/2017 protects life, health, dignity, and self-determination and establishes that no healthcare treatment may be initiated or continued without the patient’s free and informed consent, except in cases expressly provided by law [10]. These principles apply in detention. The custodial setting does not remove decision-making autonomy; rather, it requires particular attention to voluntariness, capacity, confidentiality, communication barriers, and the possible influence of institutional or peer pressure. The law also reinforces the relationship of care and trust between patient and physician, making high-quality communication a substantive component of care rather than a merely formal requirement.

- Freedom of Consent in a Coercive Environment: Consent should be obtained without pressure from healthcare staff, custodial personnel, other incarcerated persons, or family members. Where voluntariness is uncertain, the clinician should explore coercive influences, ensure privacy where feasible, use interpreters when required, and document the decision-making process.
- Comprehensive Information and Documentation: Information should cover diagnosis, prognosis, expected benefits, material risks, reasonable alternatives, and the consequences of refusal, in accordance with Law 219/2017 [10]. Documentation should record the information provided, questions raised by the patient, the decision reached, and any subsequent change in preferences. Tailored consent processes are especially important for invasive procedures, high-risk treatment, and patients with communication or cognitive difficulties.
- Language and Cultural Barriers: Foreign nationals represent a substantial share of the prison population and may face language barriers, isolation, and different health beliefs, which affect access to care, the reliability of clinical histories, and the validity of consent [16]. In Italy, cultural mediation and interpreting services in prisons depend on the Penitentiary Administration and are not consistently available to healthcare teams. In the authors’ center, clinicians use digital real-time translation tools to communicate with patients who cannot express themselves adequately, including when providing information on diagnostic and therapeutic procedures and obtaining informed consent, and their use is recorded in the clinical record. Such tools are useful but have limitations in the accuracy of medical terminology and in confidentiality; professional interpreters should therefore be prioritized for high-risk decisions, capacity assessments, and psychiatric evaluations, supported by translated written materials and attention to cultural and religious beliefs that may influence the acceptance of treatment.

### 3.3. Refusal of Treatment, Hunger Strikes, and Advance Directives (DAT)

Hunger strikes and treatment refusal represent a paradigmatic area in which clinical duties, autonomy, detention, and institutional responsibility intersect. Management should be protocolized but individualized. Law 219/2017 provides the domestic framework for informed refusal, while the World Medical Association Declaration of Malta offers specific ethical guidance for physicians caring for hunger strikers [10,11]. The central questions are decision-making capacity, voluntariness, clinical risk, and the validity and persistence of the patient’s expressed wishes.

- Capacity Evaluation: Clinicians should assess whether the patient can understand, retain, weigh, and communicate information relevant to the decision. Psychiatric consultation may be appropriate where psychosis, severe mood disorder, cognitive impairment, intoxication, withdrawal, or suicidal intent is suspected; however, psychiatric diagnosis alone does not establish incapacity.
- Documented Information and Monitoring: The patient should receive repeated, comprehensible information on the foreseeable physiological consequences of prolonged fasting, warning signs, available monitoring, and treatment options. Clinical review should be proportionate to the evolving risk and should document vital signs, hydration, metabolic status, capacity, expressed preferences, and any evidence of coercion.
- Respect for a Competent Refusal: Where an adult patient has decision-making capacity and maintains a free and informed refusal, treatment decisions should be governed by Law 219/2017 and applicable legal safeguards [10]. The WMA Declaration of Malta considers forced feeding of a mentally competent hunger striker contrary to an informed and voluntary refusal ethically unacceptable [11]. The legal and ethical analysis should therefore focus on capacity, voluntariness, emergency circumstances, and any specific statutory basis for involuntary treatment, rather than treating custodial status or a psychiatric procedure as an automatic authorization for forced intervention.
- Institutional and Judicial Communication: When a hunger strike or refusal creates a serious risk to life or substantial deterioration, healthcare leadership should ensure timely communication with the prison director and, where legally relevant, the competent judicial authority. Such communication should preserve clinical confidentiality to the greatest extent permitted by law and should document the respective responsibilities of healthcare, custodial, and judicial actors rather than transferring clinical decision-making to non-medical authorities.

## 4. Clinical Risk Management and Governance in Correctional Facilities

### 4.1. Patient Safety Behind Bars (Law 24/2017)

Law 24/2017 defines safety of care as a constitutive element of the right to health and requires healthcare organizations and professionals to participate in prevention and management of clinical risk [15]. This obligation applies fully in correctional healthcare. The relevant risks, however, are generated by both disease burden and the environment in which care is delivered. For this reason, hospital-derived risk-management tools require adaptation to prison-specific processes, including movement restrictions, security gates, escort availability, medication handling, communication between healthcare and custody, and frequent transfers.

- Structural Differences between Prisons and Hospitals: Correctional institutions are primarily custodial environments within which the NHS operates. Physical barriers, controlled movement, staffing patterns, authorization procedures, and transport requirements can affect response times and access to diagnostic or therapeutic resources [1,6,7,8]. Risk analysis should therefore map not only clinical processes but also the interfaces on which those processes depend.
- Variability and Contextual Complexity: Quality and safety indicators may be influenced by treatment refusal, transfers, release, medication diversion, self-harm, language barriers, mental illness, substance use, and interrupted follow-up. These factors should not be used to normalize lower standards; rather, they justify more robust traceability, explicit escalation criteria, and correctional-specific process indicators. The objective is to distinguish preventable system failure from clinically unavoidable variability and to identify where redesign can reduce risk.

### 4.2. Risk Mapping and Medication Governance

Key risk areas in correctional medical centers include the following:

- Medication Governance: Storage, dispensing, and administration require enhanced controls because some medications may be diverted, exchanged, accumulated, or intentionally misused. Psychotropic drugs, opioid and non-opioid analgesics, sedatives, and other high-risk medicines should be governed through locally defined risk-based procedures that balance safety with therapeutic appropriateness and patient dignity.
- Diagnostic and Therapeutic Delays in Emergencies: Acute coronary syndrome, stroke, sepsis, major trauma, and other time-dependent emergencies require protocols that minimize delay between recognition, initial stabilization, activation of emergency services, and transfer. D.P.R. 230/2000 and the broader prison-health framework provide for external healthcare when necessary; operational protocols should specify who activates transport, how security arrangements are escalated, and what clinical information accompanies the patient [6,14].
- Infectious Disease Control: The burden of blood-borne and airborne infection remains clinically important in prison populations. Prevention requires screening, vaccination, infection-control procedures, harm-reduction strategies where applicable, prompt treatment, and continuity during transfer or release. WHO monitoring continues to identify hepatitis C, tuberculosis, HIV, and continuity of care as relevant areas for prison-health improvement [8].

Mitigation strategies implemented or recommended by executive leadership include the following:

- Directly Observed Therapy and Unit-Dose Dispensing: For selected high-risk medicines or patients, supervised administration and unit-dose systems can reduce accumulation and diversion. These strategies should be applied proportionately and reviewed clinically so that security-driven medication procedures do not become a substitute for individualized prescribing. In the authors’ center, directly observed therapy is already in place, whereas unit-dose dispensing is not yet available and represents a recommended development.
- Balancing Supervision and Autonomy in Chronic Treatment: Directly observed therapy (DOT) and self-administration (“in-possession” medication) should be regarded as complementary options rather than alternatives. International guidance recommends an individual risk assessment to decide whether a person can hold medicines in possession, reviewed whenever circumstances change—for example after a medication incident, evidence of self-harm, hoarding, non-adherence, or a move to a segregation unit—while reserving mandatory directly observed administration for specific categories such as controlled drugs and anti-tuberculosis treatment [17]. For most chronic conditions, such as hypertension, diabetes, or dyslipidemia, supervised self-administration supports patient autonomy, health literacy, and the skills needed to manage treatment after release. DOT should therefore be applied proportionately, based on the medicine’s risk profile and the individual’s clinical and safety assessment, and progressively reduced when the risk allows.
- Electronic Medication Records: Digital systems linking prescribing, pharmacy preparation, dispensing, and nursing administration can reduce transcription errors, improve identification of omitted doses, support reconciliation after transfer, and create an auditable medication history. Interoperability with regional systems is particularly important where patients frequently move between prison, hospital, and community services.

### 4.3. Internal Audits, Digital Traceability, and Litigation Reduction

Ensuring clinical safety depends on continuous verification:

- Clinical Governance Audits: Multidisciplinary reviews involving physicians, nurses, specialists, and prison administrators assess adverse events, near misses, and systemic bottlenecks.
- Electronic Health Records (EHR): Correctional healthcare should use interoperable records capable of documenting assessments, prescriptions, administration, consent and refusal, external referrals, hospital discharge information, and transfer summaries. Time-stamped entries and clear authorship strengthen clinical continuity and retrospective audit. Digital traceability should be designed primarily as a patient-safety and continuity tool; its medico-legal value derives from accurate contemporaneous documentation rather than defensive documentation alone.
- Patient Feedback and Complaints: In Italy, people in custody can submit complaints, including on healthcare, to the Supervisory Magistrate, who requests information and supporting documents from the healthcare unit and may meet the patient [4]. This judicial safeguard, which is operational in the authors’ center, should be complemented by healthcare-led feedback channels—such as anonymous questionnaires and periodic patient-experience surveys—so that recurring problems are analyzed through the same audit cycle used for adverse events. Indicators for prison healthcare have so far been selected with limited involvement of people with lived experience of imprisonment, and their inclusion has been recommended [18].
- Secure Interoperability between Custodial and Health Information Systems: Prison administrative networks and regional health information systems are usually separated for security reasons, and their lack of interoperability is a recognized source of clinical risk, leading to duplicate data entry, manual transfers, transcription errors, and incomplete information on patients’ risks [19,20]. Interoperability does not require merging the two systems. A safer approach is a minimum, standardized data exchange through secure, audited interfaces: the custodial system transmits only the information needed for care (identity, location, movements, scheduled transfers, and release dates), while clinical data remain within the health information system under the control of the health authority, with role-based access and full traceability of every access. Any exchange must comply with data protection legislation, including the principles of purpose limitation and data minimization of the General Data Protection Regulation (GDPR) [21].

### 4.4. Protecting the Healthcare Workforce

Workforce instability is itself a clinical risk. Correctional healthcare professionals are exposed to verbal and physical aggression, high workloads, perceived danger, and repeated contact with self-harm and trauma, factors associated with burnout and emotional exhaustion [22,23]. In the authors’ experience, nursing staff are the most affected professional group, and the occupational physician responsible for fitness-for-work surveillance plays an important role in identifying and addressing staff needs. Organizational protection should include structured debriefing after critical events (e.g., suicide attempts, aggression, or deaths in custody); access to clinical supervision and psychological support; workload and shift planning that avoids chronic understaffing; procedures for the prevention and reporting of workplace violence agreed with custodial staff; and training and career pathways that recognize correctional healthcare as a specialized field. Mindfulness-based and psychoeducational programs have shown promising effects on burnout in correctional settings, although the evidence remains limited [22].

## 5. Operational and Managerial Models of the Prison Medical Center

### 5.1. Internal Organization: Triage, Intake, and Risk Stratification

To optimize resource allocation, admissions and stays in the Prison Medical Center follow a structured triage system and stratification framework:

- Multidisciplinary Intake: Admission involves integrated evaluations by medical, nursing, psychiatric, and social work staff.
- Stratification by Clinical Complexity.
- Low Complexity/Short-Term Stay: Acute conditions needing brief observation or post-procedural monitoring.
- Moderate to High Clinical Complexity: Decompensated chronic conditions, active oncology care, or severe infectious diseases.
- Integrated Complexity (Mental Health and Substance Use): Dual-diagnosis cases are managed collaboratively by medical staff, addiction services (Ser.D), and Mental Health Departments (DSM). This approach helps ensure that the center remains focused on clinically defined needs while patients whose principal needs are social, custodial, or behavioral are directed to the most appropriate multidisciplinary pathway.

### 5.2. Dedicated Care Pathways (PDTAs)

Re-engineering necessitates the adaptation of clinical pathways (PDTAs) to correctional contexts:

- Infectious Diseases: Structured pathways should provide systematic testing, confirmatory diagnostics, treatment initiation, monitoring, and continuity planning for HCV, HIV, tuberculosis, and other relevant infections. Evidence indicates that prison-based HCV treatment can achieve outcomes comparable to community treatment when care is completed, while transfer or release during therapy can undermine outcomes if continuity is not actively managed [24]. More recent evidence also supports organized interventions to improve testing, linkage to care, and Direct-acting antiviral (DAA) treatment initiation in prison settings [25].
- Mental Health and Suicide Prevention: Given the high burden of mental illness and suicide risk described in European prison-health data, pathways should integrate intake screening, structured risk assessment, timely psychiatric and psychological care, substance-use services, multidisciplinary case review, and transition planning [8]. Custodial staff can contribute to recognition and communication of behavioral change, but diagnosis, treatment, and clinical risk decisions should remain under healthcare responsibility.
- Chronic Disease and Disability: Tailored care plans for elderly or mobility-impaired inmates encompass barrier-free accommodations, prosthetic aids, and scheduled follow-ups by specialists.

**Figure 1:**
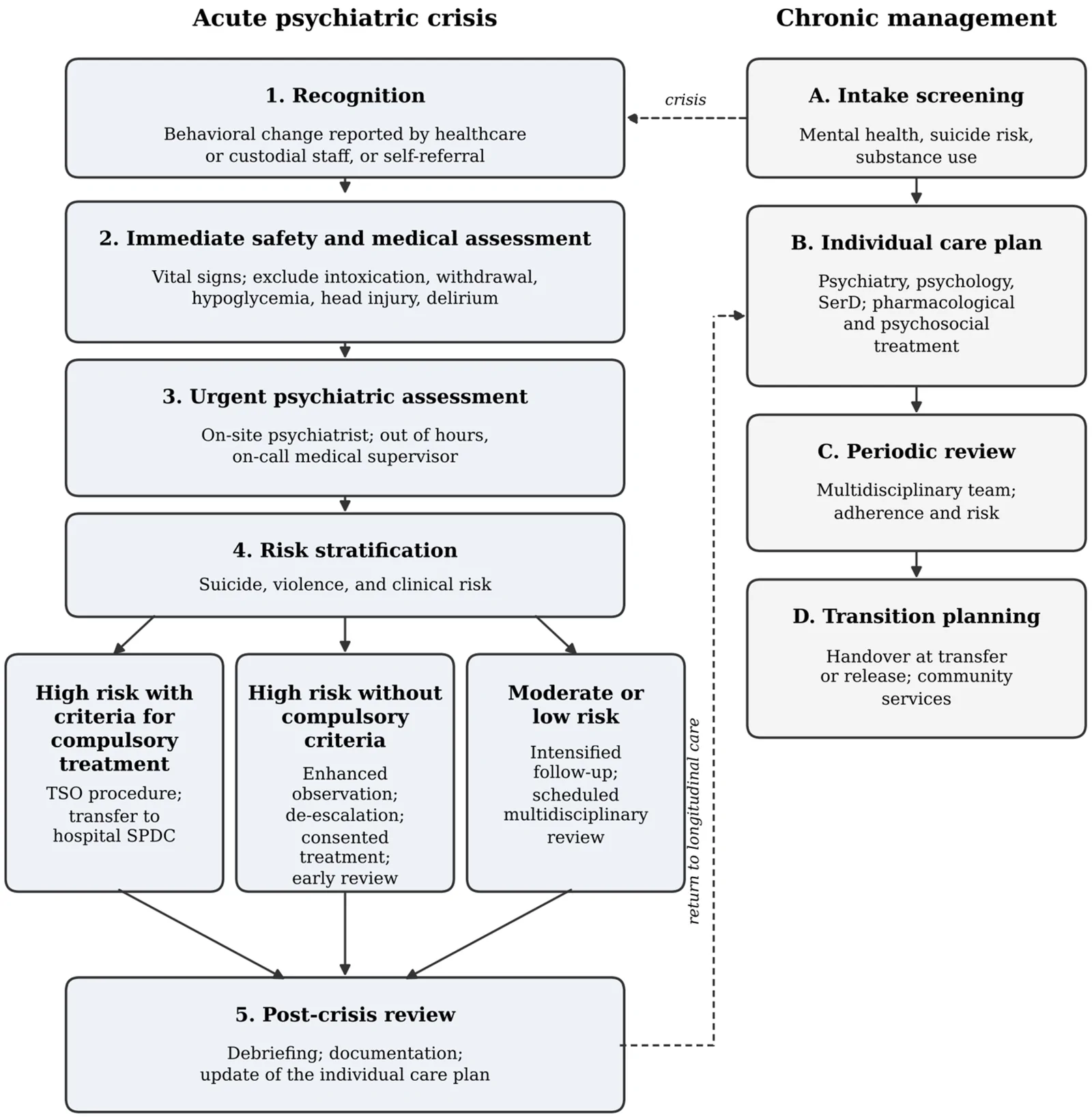
Proposed stepwise pathway for acute psychiatric crises and longitudinal mental health management in a prison medical center. SerD: addiction services; SPDC: Psychiatric Diagnosis and Treatment Service; TSO: compulsory health treatment.

Mental health care in particular requires a clear distinction between the management of acute crises and longitudinal care (Figure 1). In the authors’ center, a dedicated team of three full-time psychiatrists and eight psychologist-psychotherapists provides on-site care, while compulsory treatment, when required, follows the national procedure for compulsory health treatment (TSO) [26] and entails transfer to the hospital Psychiatric Diagnosis and Treatment Service (SPDC). The proposed pathway is consistent with international recommendations on mental health care for people in contact with the criminal justice system [27].

### 5.3. Interface with Protected Hospital Units and Care Continuity

When tertiary hospital care is necessary (e.g., complex surgeries, advanced diagnostics, ICU admission), executive management coordinates transitions across interfaces:

- Protected Hospital Wards: Specialized, secure units within public NHS hospitals extend the prison medical center’s capabilities, facilitating planned admissions and timely discharges back to the facility. Where protected wards are not available, detained patients may also be admitted to ordinary hospital wards under the surveillance of the Penitentiary Police [6].
- Rationalization of External Transfers: Point-of-care testing, digital ECG, focused bedside ultrasound where appropriately governed, and teleconsultation can reduce transfers that do not add clinical value, while accelerating specialist input. The objective is not to reduce external referrals indiscriminately; patients must still be transferred whenever the required diagnostic or therapeutic standard cannot be safely achieved inside the institution. Evidence on prison telehealth suggests potential benefits for access and process efficiency, although outcomes depend on local implementation and should be monitored [28,29].
- Discharge Planning and Community Continuity: Before release or transfer, healthcare teams should reconcile medication, provide an updated clinical summary, identify pending investigations, arrange referral to primary or specialist care, and provide a sufficient medication bridge where permitted. For mental illness and other complex conditions, evidence indicates that interventions beginning before release can improve contact with community health services, although effects on reoffending are heterogeneous and should not be overstated [12,13].

### 5.4. The Nursing Contribution

Nurses provide the most continuous clinical presence in correctional healthcare, and many of the processes described in this commentary depend on them. Their contribution includes early detection of clinical deterioration and behavioral change through continuous observation; medication safety, including preparation, directly observed administration, monitoring of adherence, and reconciliation after transfer; chronic disease management through monitoring, education, and support for self-management; health education and prevention; participation in intake assessment, suicide-risk screening, and multidisciplinary review; and discharge planning, including medication bridges and handover to community services. In the authors’ center, the nursing coordinator also oversees the pharmacy, directly linking nursing practice and medication governance. Nurse-led review of medication incidents and near misses, nursing involvement in the design of shared procedures, and dedicated training are therefore integral components of the proposed model.

## 6. Innovation, Telemedicine, and Future Perspectives

### 6.1. Digitalization and Telemedicine

Digital technologies can mitigate specific structural barriers in correctional healthcare, particularly where specialist availability, transport, geography, or security procedures delay access. They should be introduced as components of clinical pathways rather than as stand-alone technological solutions. Systematic reviews indicate that telehealth can improve access and may produce clinical outcomes comparable to conventional care in selected contexts, but the evidence base remains heterogeneous and implementation quality is decisive [28,29].

- Tele-consultations: Secure video consultations can support specialties such as dermatology, cardiology, endocrinology, infectious diseases, neurology, pain medicine, and psychiatry. Appropriate use requires criteria for in-person escalation, privacy, reliable connectivity, access to relevant examination data, and integration of specialist recommendations into the local health record.
- Decentralized Diagnostics: Point-of-care laboratory testing, digital ECG with remote interpretation, and other validated near-patient technologies can shorten diagnostic pathways and support timely triage. Their use requires quality assurance, staff training, maintenance, calibration, and clear responsibility for acting on results.
- Tele-psychiatry and Longitudinal Follow-Up: Virtual care can support specialist review, continuity after internal transfer, and selected follow-up pathways, but it should not replace face-to-face assessment when physical presence is clinically necessary. Digitalization may reduce travel and security burden while increasing continuity, yet privacy, data protection, informed participation, and equitable access must be treated as core design requirements [28,29].
- Data Protection, Consent, and Infrastructure: Telemedicine in custody raises specific challenges. Confidentiality may be compromised when consultations take place in rooms accessible to custodial staff or when escorts are present; dedicated spaces, defined criteria for the presence of staff, and documentation of who was present are therefore required. Consent to remote consultation should be informed and voluntary, and the patient should be able to request an in-person assessment where clinically appropriate. Data processing must comply with the GDPR [21], with encrypted connections, secure storage, and a clear allocation of data-controller responsibilities between the health authority and any external provider. Finally, infrastructure in older prison buildings—bandwidth, connectivity in clinical areas, and the authorization of devices inside secure perimeters—is often the main practical barrier, and its assessment should precede any telemedicine program [30].

### 6.2. Gender-Specific Medicine in Correctional Facilities

Women remain a small minority of the Italian prison population, representing approximately 4.5% of people in custody as of 30 June 2026 (2891 of 64,773) [31]. Their numerical minority should not result in care pathways designed primarily around male populations. Contemporary evidence indicates that women in prison have substantial burdens of communicable disease, mental-health conditions, substance-use disorders, reproductive-health needs, and trauma exposure [32,33]. Gender-responsive care should therefore be treated as an organizational requirement rather than an optional service.

- Reproductive Health and Screening: Cervical-cancer screening, HPV-related prevention, breast screening, contraception, menstrual health, sexual health, and management of gynecological conditions should follow national standards and individual risk. Access should be equivalent to community pathways, with appropriate privacy and continuity when women are transferred or released.
- Maternal and Child Health: Pregnant women require antenatal, obstetric, perinatal, and postnatal care consistent with community standards, with particular attention to mental health, substance use, medication safety, safeguarding, and continuity. Where mother–child arrangements are used, health planning should address the needs of both mother and child and should be coordinated with social and pediatric services. When young children live with their mothers in custody, which in Italy occurs mainly in low-security institutions for detained mothers (ICAM) [34], healthcare planning should also include pediatric follow-up and vaccination, developmental and psychological monitoring, support for the mother–child relationship, and protocols that limit the child’s exposure to security procedures. International standards require that the health of accompanying children be assessed by a specialist and that their best interests guide all decisions [35]. Available evidence suggests that mother–child programs may support attachment, but it remains limited and heterogeneous, so these arrangements should be carefully evaluated [36].
- Trauma-Informed Care: Histories of trauma and post-traumatic stress are common in incarcerated populations and may be particularly prevalent among women [32,33]. Trauma-informed practice does not imply assuming that every patient has a trauma disorder; it means organizing communication, examination, mental-health pathways, and safeguarding in ways that minimize re-traumatization and preserve autonomy, privacy, and dignity.

The organizational response should be proportionate to the size of the female population. Where women are few and dispersed across mixed institutions, as in Italy, the main risks are isolation, limited access to specialist care, and pathways designed around men; these can be addressed through regional referral to designated gynecology and obstetrics services, scheduled outreach clinics, telemedicine for selected follow-up, and a named healthcare professional responsible for women’s health in each institution. Where the female population is larger or concentrated in dedicated institutions, on-site gynecological, perinatal, and trauma-informed mental-health services become feasible. In both cases, female healthcare staff should be available on request for examinations, and pathways should be monitored through sex-disaggregated indicators [32,35].

### 6.3. Rehabilitative Value of Healthcare (Article 27 of the Constitution)

Health protection in prison is consistent with the constitutional rehabilitative purpose of punishment [37] and with the broader public-health principle that people in custody eventually return to the community. The contribution of healthcare to rehabilitation should nevertheless be described in clinical and public-health terms rather than as a guarantee of behavioral outcomes. Three domains are particularly relevant:

- Conflict Reduction and Institutional Climate: Timely access, transparent communication, and predictable healthcare procedures can reduce uncertainty and disputes surrounding medical requests. Any effect on institutional safety should be evaluated locally rather than assumed.
- Health Literacy and Prevention: Detention can provide an opportunity to diagnose previously untreated conditions, update vaccination, initiate treatment, address substance use, and improve understanding of chronic disease and medication. This opportunity is especially important for people who had limited access to healthcare before incarceration [7,8].
- Continuity After Release: Maintaining treatment for psychiatric, substance-use, infectious, and chronic conditions after release is clinically important and may improve engagement with community services. Evidence on effects on reoffending or reincarceration is heterogeneous; therefore, continuity of care should be justified primarily by health need, patient safety, and equity rather than by assuming a uniform criminological effect [12,13].

## 7. Conclusions and Recommendations

### 7.1. From Problem Diagnosis to Systemic Re-Engineering

The question of whether penitentiary medicine “exists” in Italy is now better reframed as a question of operational maturity. The legal basis for healthcare in prison is established, but quality depends on whether clinical, organizational, digital, and medico-legal processes are translated into reliable everyday practice (Table 2). Experience from prison medical-center leadership suggests that many improvements can be pursued within the existing statutory framework through clearer governance, standardized procedures, digital traceability, multidisciplinary pathways, and stronger integration with regional health services. This does not exclude the possibility that future legislative or regulatory reform may be useful; rather, it emphasizes that substantial quality improvement is achievable through more complete implementation of current obligations [2,3,4,5,6,7,8,9,10,14,15].

### 7.2. Actionable Recommendations

Based on the operational domains analyzed above, four priorities are proposed for healthcare authorities and prison administrations. These recommendations are intentionally organizational: they are intended to make existing clinical and legal standards more consistently executable and auditable across institutions.

1. Standardize Prison Medical Centers Nationally: Define minimum structural, technological, staffing, emergency-response, and clinical-governance standards for prison medical centers, while allowing adaptation to regional networks and institutional size.
2. Formalize Shared Standard Operating Procedures: Establish jointly agreed NHS–Penitentiary Administration procedures for emergency activation, movement for clinical care, medication processes, external transfer, return from hospital, management of treatment refusal, and communication of time-critical clinical risk. SOPs should specify responsibilities without blurring clinical and custodial authority.
3. Expand Interoperable Electronic Health Records: Develop or complete integration between prison healthcare records, regional NHS systems, prescribing platforms, and hospital discharge information so that transfers do not create avoidable information gaps. Auditability, access control, confidentiality, and continuity should be built into system design.
4. Support Specialized Professional Training: Provide targeted training in correctional medicine, clinical risk management, mental health, addiction medicine, infectious disease, informed consent, dual-loyalty ethics, trauma-informed practice, and interprofessional communication. Training should involve both healthcare and, for interface procedures, relevant custodial staff while preserving professional role boundaries.

To make these recommendations auditable, each can be associated with a limited set of indicators (Table 3). The indicators are proposed as examples and should be selected locally according to available data systems, combining process, outcome, and patient-reported measures, as recommended for prison healthcare [18].

### 7.3. Adaptability, Resource Implications, and Transfer to Other Jurisdictions

The framework was developed within the Italian legal system, but its core principles—equivalence of care, clinical independence, patient autonomy, safety of care, and continuity—are shared by international standards, including the United Nations Standard Minimum Rules for the Treatment of Prisoners (Nelson Mandela Rules) [38] and the European Prison Rules [9]. Adaptation to other jurisdictions therefore requires distinguishing these principles from the national instruments used to implement them. Italian Laws 24/2017 and 219/2017 are, respectively, domestic expressions of the duty to manage clinical risk and of the right to informed consent and refusal [10,15]; in other countries, the corresponding obligations may derive from different legislation, case law, or professional regulation, but the operational questions remain the same: who assesses capacity, how refusal is documented, when judicial authorities must be informed, and how responsibility is shared between healthcare and custody. With regard to hunger strikes, the WMA Declaration of Malta provides a common ethical reference that applies regardless of national law [11]. Similarly, the allocation of authority for external transfers summarized in Table 1 is specific to Italy, but every system needs an equivalent map of who authorizes movement for clinical reasons, including outside office hours.

Implementation also has resource implications. Prison health expenditure is reported by few countries and in non-comparable ways [39], and smaller or underfunded health authorities may not be able to establish dedicated medical centers or advanced digital systems. The framework is therefore modular: shared procedures, structured intake, risk-based medication governance, and documented consent processes require mainly organizational investment, whereas electronic interoperability, point-of-care diagnostics, and telemedicine require dedicated funding. In Italy, this need is already addressed by the hub-and-spoke organization of regional prison health networks defined by the 2015 Unified Conference Agreement [6]: within each region, institutions with an internal medical center (SAI) act as hubs for institutions that provide only primary care, while hospital care is delivered in reserved areas or protected wards of public hospitals, or in ordinary wards under the surveillance of the Penitentiary Police. A comparable network model, supported by teleconsultation, shared protocols, and planned admissions, may allow other jurisdictions with limited resources to provide access to higher-intensity care without replicating it in every institution.

### 7.4. An Ethical, Medico-Legal, and Social Imperative

In conclusion, the re-engineering of correctional healthcare addresses a threefold requirement:

- Clinical and healthcare-related: To ensure health protection and care efficiency in accordance with Article 32 of the Constitution [37].
- Medico-legal and safety-oriented: To strengthen patient safety, professional accountability, traceable decision-making, and the defensibility of clinical processes, while reducing preventable harm and avoidable disputes.
- Ethical and constitutional: To uphold dignity, autonomy, equivalence of care, and the rehabilitative framework established by the Italian Constitution. A mature correctional health system is one in which deprivation of liberty does not become deprivation of clinically appropriate care and in which the institutional response to complexity is better organization rather than acceptance of lower standards.

## Figures and Tables

**Table 1:** Competent authority for transfers to external healthcare facilities under Article 11(4) of Law 354/1975 [4,5].

Procedural Status/Stage	Competent Authority for Transfer to an External Healthcare Facility
Before exercise of criminal action	Judge for Preliminary Investigations (GIP)
Proceedings before a single judge	Judge conducting the proceedings
Proceedings before a collegiate court	President of the judicial panel
Direct trial before presentation for validation of arrest in flagrante delicto	Public Prosecutor
Convicted persons and internees	Supervisory Magistrate

Note: In cases of extreme urgency (“estrema urgenza”), Article 17 of Presidential Decree 230/2000 allows the immediate transfer of the detained person to an external healthcare facility, at any time of day or night, on the basis of a medical certification issued by the physician on duty in the institution; the prison administration subsequently informs the competent judicial authority for ratification [14]. The judicial authorization summarized in the table therefore concerns non-urgent transfers.

**Table 2:** Operational re-engineering framework for correctional healthcare.

Operational Domain	Critical Interface or Risk	Re-Engineering Intervention	Primary Quality Objective
Governance	Fragmented NHS–custody responsibilities	Shared SOPs, escalation rules, explicit responsibility mapping	Timeliness, accountability, clinical independence
Admission and triage	Incomplete intake information and heterogeneous risk	Multidisciplinary intake, structured clinical and suicide-risk screening	Early identification and appropriate allocation
Medication governance	Diversion, accumulation, omitted doses, reconciliation failures	Risk-based supervised administration, unit-dose processes, electronic medication records	Medication safety and traceability
Emergency care	Delay caused by movement, escort, authorization, or transport interfaces	Time-critical pathways with predefined clinical and custodial activation steps	Reduced avoidable delay
External specialist care	Overuse or delayed use of hospital transfer	Internal diagnostics, teleconsultation, explicit referral and transfer criteria	Appropriate setting of care
Consent and refusal	Coercion, uncertainty about capacity, poor documentation	Private communication, capacity assessment, repeated information, auditable documentation	Autonomy and medico-legal robustness
Infectious and chronic disease	Interrupted treatment during transfer or release	Dedicated PDTAs, medication reconciliation, transfer and discharge summaries	Continuity and treatment completion
Digital health	Fragmented records across prison, hospital, and community	Interoperable EHR, digitally traceable prescriptions and referrals	Continuity, auditability, patient safety
Gender-responsive care	Male-centered pathways and under-recognition of trauma/reproductive needs	Gender-specific prevention, reproductive care, trauma-informed pathways	Equity and person-centered care
Release planning	Loss to follow-up and gaps in medication or specialist care	Pre-release referral, medication bridge, community handover and follow-up plan	Continuity across the prison–community transition

**Table 3:** Example indicators for monitoring the proposed recommendations.

Recommendation	Example Indicators
1. Standardize Prison Medical Centers	Proportion of centers meeting defined minimum standards; median time from clinical request to specialist assessment; number of emergency simulations performed per year
2. Formalize shared SOPs	Median time from recognition of an emergency to activation of external transport; proportion of external transfers delayed for non-clinical reasons; proportion of treatment refusals with documented capacity assessment
3. Expand interoperable EHRs	Proportion of transfers with a clinical summary available on arrival; proportion of medication reconciliations completed within 24 h of admission or transfer; proportion of releases with a discharge summary and medication bridge
4. Support specialized training	Proportion of staff completing core training annually, including dual-loyalty ethics; staff turnover; proportion of complaints resolved within the defined time

## Data Availability

No new data were created or analyzed in this study. Data sharing is not applicable to this article.

## Abbreviations

The following abbreviations are used in this manuscript:

LEA	Essential Levels of Care
SAI	Intensive Assistance Service (Servizio di Assistenza Intensificata)
WHO	World Health Organization
NHS	National Health Service
DAP	Department of Penitentiary Administration
SOP	Standard Operating Procedure
EHR	Electronic Health Record
PDTA	Diagnostic-Therapeutic Care Pathway
DOT	Directly Observed Therapy
GDPR	General Data Protection Regulation
ICAM	Low-Security Institution for Detained Mothers (Istituto a Custodia Attenuata per Madri)
SerD	Addiction Services
SPDC	Psychiatric Diagnosis and Treatment Service (Servizio Psichiatrico di Diagnosi e Cura)
TSO	Compulsory Health Treatment (Trattamento Sanitario Obbligatorio)
